# COVID-19 Lockdown Increased the Risk of Preterm Birth

**DOI:** 10.3389/fmed.2021.705943

**Published:** 2021-09-27

**Authors:** Ting-ting Lin, Chen Zhang, Lei Chen, Li Jin, Xian-hua Lin, Jie-xue Pan, Cindy-Lee Dennis, Ben W. Mol, He-feng Huang, Yan-ting Wu

**Affiliations:** ^1^Shanghai Key Laboratory of Embryo Original Diseases, The International Peace Maternity and Child Health Hospital, School of Medicine, Shanghai Jiao Tong University, Shanghai, China; ^2^Research Department, Institute of Reproduction and Development, Obstetrics and Gynecology Hospital, Fudan University, Shanghai, China; ^3^Lawrence S. Bloomberg Faculty of Nursing, University of Toronto, Toronto, ON, Canada; ^4^Monash University Monash Medical Centre, Clayton, VIC, Australia; ^5^Aberdeen Centre for Women's Health Research, University of Aberdeen, Aberdeen, United Kingdom

**Keywords:** COVID-19, lockdown, preterm birth, very preterm birth, premature rupture of membranes

## Abstract

**Purpose:** To estimate whether the city-specific lockdown in Shanghai induced by the COVID-19 pandemic affected preterm birth rates among uninfected pregnant women in different trimesters.

**Methods:** The population-based retrospective cohort study was conducted in the International Peace Maternity and Child Health Hospital (IPMCH) in Shanghai, China. Pregnant women without COVID-19 received perinatal healthcare during lockdown (from January 24, 2020 to March 24, 2020) and non-lockdown (from January 24, 2019 to March 24, 2019) period and giving birth to a live infant at IPMCH were enrolled. 1:1 propensity score matching and Inverse probability of treatment weighting were used to evaluate preterm birth (<37 weeks), very preterm birth (<34 weeks), preterm birth with premature rupture of membranes (PROM-PTB), spontaneous preterm birth with intact membranes (S-PTB), and medically induced preterm birth (MI-PTB) between two groups.

**Results:** 8,270 pregnant women were in the lockdown group, and 9,815 were in the non-lockdown group. Pregnant women in second trimester during lockdown had a higher risk of PTB than those during the non-lockdown period [OR: 1.43 (CI 1.01–2.02), ARD: 1.7% (CI 0.04–3.4%), *p* = 0.045]. Furthermore, pregnant women in third trimester during lockdown had a higher risk of PROM-PTB than those during the non-lockdown period [OR: 1.64 (CI 1.09–2.47), ARD: 0.9% (CI 0.2–1.6%), *p* = 0.02]; no group differences were found related to rates of VPTB, S-PTB or MI-PTB.

**Conclusion:** In this cohort study in China, we found that there was an increased risk in preterm birth for non-infected women in COVID-19 lockdown who were in their second trimester.

## Introduction

Coronavirus disease 2019 (COVID-19) is a highly infectious disease with a significant mortality rate that has limited effective treatment options (1). In late 2019, hospitals in Wuhan City in Hubei Province admitted multiple patients with unexplained pneumonia with a history of exposure to the South China Seafood Market, and these cases of pneumonia have since been confirmed as COVID-19. COVID-19 was officially identified as a type B infectious disease with human-to-human transmission by the National Health Council on January 20, 2020 (2). Due to the high contagiousness of COVID-19 and the population's general susceptibility, a city-wide lockdown was declared in Shanghai on January 24, 2020 and ended on March 24 (3), 2020. During the lockdown, (1) childcare facilities, schools and universities were closed; (2) all non-emergency workers were required to work from home; (3) gatherings of more than 10 people were banned; and (4) industrial production was almost at a standstill (3). Rates of anxiety and depression increased rapidly among the general population (4, 5).

Preterm birth (PTB) is a significant cause of infant mortality and morbidity (6). Infants born preterm are at higher risk for various health problems and developmental delays, often resulting in emotional and financial difficulties for families and an increased use of health and social services (7). Previous research suggests that societal lockdown procedures implemented by governments to mitigate the impact of the COVID-19 pandemic on population health may result in reduced rates of preterm birth, particularly among those occurring at very low gestational ages (8–10). In a US study examining hospital birth data, there was a significant decrease in the incidence of PTB in 2020 during the COVID-19 pandemic compared with the rates during the 2019 pre–COVID-19 period (9.9 vs. 12.6%; OR, 0.76; 95% CI, 0.58–0.99). There was also a significant decrease in PTB at <34 weeks gestation (2.5 vs. 4.7%; aOR, 0.51; 95% CI, 0.31–0.82) and PTB at <28 weeks gestation (0.6 vs. 1.5%; aOR, 0.37; 95% CI, 0.15–0.93) in 2020 compared with the rates in 2019 (9). In an Irish study where regional data covering most of the 2020 lockdown period were compared with historical regional and national data, an unprecedented reduction in births of very low birthweight (VLBW) and extremely low birthweight (ELBW) infants was observed coinciding with the COVID-19 lockdown (10). In a national quasi-experimental study conducted in the Netherlands, the initial implementation of COVID-19 mitigation measures in early March 2020 was associated with a substantial reduction in the incidence of preterm births in the following months (8). Although these studies have reached similar conclusions, they only compared the outcomes among pregnant women in their third trimester during the lockdown period. It remains unknown whether the pregnancy outcomes of women in their first or second trimester are affected by the COVID-19 lockdown.

The purpose of this study was to follow uninfected pregnant women in all three trimesters of pregnancy during the COVID-19 lockdown in Shanghai to determine whether the lockdown independently affected PTB rates.

## Materials and Methods

### Patient Enrollment

This retrospective cohort study was conducted at the International Peace Maternity and Child Health Hospital (IPMCH), which is a university-affiliated hospital in which ~20% of all births in Shanghai, China. Eligible pregnant women were those who had negative nasopharyngeal swab nucleic acid test when they attended routine obstetrics clinic during pregnancy and at time of delivery (11); and who received perinatal healthcare during the lockdown period. Furthermore, they had to have given birth to an infant with a gestational age >20 weeks at IPMCH. Women were excluded if they underwent *in vitro* fertilization, had a twin or multiple birth and were not of the Han race. The start of the lockdown in Shanghai was officially declared on January 24, 2020 (3), and the city-specific lockdown ended on March 24, 2020, when the Shanghai government adjusted the public health emergency response from level one to level two (12).

### Data Collection

For all eligible pregnant women, the following data were collected by hospital staff and recorded in the electronic medical file: maternal age, marital status, pre-pregnancy body weight and height, smoking status, current alcohol consumption, maternal education level, parity, preterm birth history, pregnancy complications, date of last menstrual period (LMP), paternal age, paternal education level, neonatal birth weight, Apgar score, and electronic fetal heart rate monitoring records. Maternal and paternal educational levels were defined as the years of education after graduation from primary school and were categorized as <6 years (low), 6 to 10 years (middle) and >10 years (high). Height and pre-pregnancy weight were used to obtain BMI scores which calculated by dividing the weight obtained above by the square of the height and categorized as low weight (<18.5), normal weight (18.5–23.9), overweight or obese (≥24.0). Marital status, smoking status and current consumption of alcohol were self-reported and categorized as yes or no. Parity was defined as the number of previous pregnancies resulting in at least one live birth, and was categorized as nulliparous (None) and multiparous (≥1). Preterm birth history was defined as a history of one or more live births at <37 weeks of gestation. The gestational age of all eligible women was estimated according to the date of the last menstrual period and was adjusted according to routinely performed ultrasonography in the first trimester. The gestational phase during the lockdown period induced by the COVID-19 pandemic was defined as (1) first trimester (pregnant women who were in their first trimester during the lockdown period); (2) first to second trimester (pregnant women who were in their first trimester when the lockdown started and were in second trimester when the lockdown ended); (3) second trimester (pregnant women who were in their second trimester during the lockdown period); (4) second to third trimester (pregnant women who were in their second trimester when the lockdown started and were in their third trimester when the lockdown ended); and (5) third trimester (pregnant women who were in their third trimester during the lockdown period) (**Figure 2**).

### Diagnostic Criteria

In this study, preterm birth (PTB) was defined as a birth before 37 weeks of gestation. Very early preterm birth (VPTB) was defined as a birth before 34 weeks of gestation. PTB was classified into spontaneous PTB and medically induced preterm birth (MI-PTB) according to clinical presentation, and spontaneous PTBs were further categorized as preterm birth with premature rupture of membranes (PROM-PTB) or spontaneous preterm birth with intact membranes (S-PTB). The classification criteria were as follows: (1) PROM-PTB was defined as PTB with spontaneous rupture of the membranes at <37 weeks of gestation and before the onset of contractions; (2) S-PTB was defined as spontaneous PTB with intact membranes; and (3) MI-PTB was defined as PTB after labor induction or cesarean delivery for maternal or fetal indications (13). Pregnancy complications included gestational diabetes and pregnancy-induced hypertension. Gestational diabetes mellitus (GDM) was defined as diabetes diagnosed in the second or third trimester of pregnancy that was not clearly identified as overt diabetes prior to gestation (14). Pregnancy-induced hypertension (PIH) was defined as systolic blood pressure (SBP) >140 mmHg and diastolic blood pressure (DBP) >90 mmHg during pregnancy and was classified into three conditions: (1) gestational hypertension and preeclampsia (PE); (2) pre-existing hypertension plus superimposed gestational hypertension with proteinuria; and (3) unclassifiable hypertension during pregnancy (15). Stillbirth was defined as a baby born with no signs of life at a gestational age of 24 weeks or more. Abnormal Apgar score was defined as a 5-min Apgar score of 7 or less. Fetal distress, also named as “non-reassuring fetal status” by the recommendation of ACOG Committee on Obstetrics Practice, was defined as a pathophysiological condition in which fetus was suffering from insufficient oxygen supply, which was diagnosed through electronic fetal heart rate (FHR) monitoring (16). Low birth weight (LBW) was defined as newborn whose birth weight was <2,500 g. Very low birth weight (VLBW) was defined as newborn whose birth weight was <1,500 g. Macrosomia was defined as newborn whose birth weight was more than 4,000 g.

### Statistical Analyses

Considering the differences in baseline characteristics between the two groups of participants (Table 1), we used propensity score matching to generate a cohort with similar baseline characteristics. The propensity score is the conditional probability of having a specific exposure level (lockdown vs. non-lockdown) under a given set of baseline covariates (17, 18). A multivariate logistic regression model was used to estimate propensity scores, and all baseline characteristics listed in Table 1 were included as covariates (1) using a 1:1 matching protocol with no replacement for matching with a caliper width equal to 0.005 and (2) evaluating the *P*-values of all baseline covariates before and after matching to assess whether they were balanced. Standard differences of <5% indicated a relatively small imbalance. There were no missing data for the variables in the matched cohort.

**Table 1:** Baseline characteristics total sample, unweighted sample, propensity score-matched sample, and inverse probability of treatment-weighted sample.

	**Unweighted Sample**			**Propensity 1:1 Matching**			**Inverse probability of treatment weighting**		
	**Lockdown (*N* = 8,270)**	**Non-lockdown (*N* = 9,815)**	***P*** **-value**	**Lockdown (*N* = 8,268)**	**Non-lockdown (*N* = 8,268)**	***P*** **-value**	**Lockdown (*N* = 18,088)**	**Non-lockdown (*N* = 18,084)**	***P*** **-value**
**Maternal Demographics**									
Age, median (Q1-Q3), y	31 (29–34)	31 (29–34)	<0.001	31 (29–34)	31 (29–34)	0.06	31 (29–34)	31 (29–34)	<0.001
**Marital Status**									
Married	8,220 (99.4)	9,709 (98.9)	0.001	8,218 (99.4)	8,218 (99.4)	1.00	17,920 (99.1)	17,927 (99.1)	0.54
Single or divorced	50 (0.6)	106 (1.1)		50 (0.6)	50 (0.6)		168 (0.9)	157 (0.9)	
**Body mass index, median**									
(Q1–Q3),kg/m^2^	20.8 (19.3–22.8)	20.7 (19.2–22.6)	0.009	20.8 (19.3–22.8)	20.8 (19.3–22.8)	0.08	20.8 (19.3–22.7)	20.8 (19.3–22.7)	<0.001
**Body mass index**									
Low	1,106 (13.4)	1,381 (14.1)	0.02	1,106 (13.4)	1,188 (14.4)	0.07	2,530 (14.0)	2,458 (13.6)	0.25
Normal	5,863 (70.9)	7,026 (71.6)		5,863 (70.9)	5,857 (70.8)		12,808 (70.8)	12,947 (71.6)	
Overweight or Obesity	1,301 (15.7)	1,408 (14.3)		1,299 (15.7)	1,223 (14.8)		2,751 (15.2)	2,678 (14.8)	
**Current smoker (yes)**	31 (0.4)	51 (0.6)	0.04	33 (0.4)	42 (0.5)	0.30	106 (0.6)	96 (0.5)	0.48
**Current alcohol consumption (yes)**	63 (0.8)	254 (2.6)	<0.001	63 (0.8)	69 (0.8)	0.60	319 (1.8)	317 (1.8)	0.94
**Education level**									
Low	610 (7.4)	743 (7.6)	<0.001	610 (7.4)	579 (7.0)	0.27	1,447 (8.0)	1,288 (7.1)	<0.001
Middle	5,655 (68.4)	6,972 (71.0)		5,655 (68.4)	5,749 (69.5)		12,442 (68.8)	12,762 (70.6)	
High	2,005 (24.2)	2,100 (21.4)		2,003 (24.2)	1,940 (23.5)		4,200 (23.2)	4,034 (22.3)	
**Parity**									
Nulliparous	5,763 (69.7)	6,789 (69.1)	0.43	5,761 (69.7)	5,730 (69.3)	0.60	12,548 (69.4)	12,547 (69.4)	0.99
Multiparous	2,507 (30.3)	3,029 (30.9)		2,507 (30.3)	2,538 (30.7)		5,541 (30.6)	5,537 (30.6)	
**Preterm birth history (yes)**	13 (0.2)	55 (0.6)	<0.001	13 (0.2)	6 (0.1)	0.11	28 (0.2)	39 (0.2)	0.18
**Gestational phase during COVID-19 lockdown**									
First trimester	280 (3.4)	325 (3.3)	0.001	280 (3.4)	268 (3.2)	0.73	620 (3.4)	592 (3.3)	<0.001
First to second trimester	2,088 (25.2)	2,403 (24.5)		2,088 (25.3)	2,060 (24.9)		4,630 (25.6)	4,389 (24.3)	
Second trimester	1,313 (15.9)	1,735 (17.7)0		1,313 (15.9)	1,367 (16.5)		2,888 (16.0)	3,181 (17.6)	
Second to third trimester	1,942 (23.5)	2,414 (24.6)		1,941 (23.5)	1,968 (23.8)		4,234 (23.4)	4,454 (24.6)	
Third trimester	2,647 (32.0)	2,938 (29.9)		2,646 (32.0)	2,605 (31.5)		5,716 (31.6)	5,467 (30.2)	
**Paternal demographics**									
Age, median (Q1–Q3), y	32 (30–35)	32 (29–35)	<0.001	32 (30–35)	32 (29–35)	0.06	32 (29–35)	32 (29–35)	<0.001
**Education level**									
Low	609 (7.4)	718 (7.3)	0.002	609 (7.4)	597 (7.2)	0.07	1,411 (7.8)	1,263 (7.0)	0.001
Middle	5,482 (66.3)	6,734 (68.6)		5,482 (66.3)	5,617 (67.9)		12,050 (66.6)	12,349 (68.3)	
High	2,179 (26.3)	2,363 (24.1)		2,177 (26.3)	2,054 (24.8)		4,627 (25.6)	4,473 (24.7)	

Inverse probability of treatment weighting (IPTW) was used to investigate the COVID-19 exposure among the entire population of pregnant women when this population hypothetically moved from no COVID-19 exposure (non-lockdown) to exposure (lockdown) in different pregnancy phases. Participants were weighted by the inverse of the probability of exposure to the COVID-19 lockdown. The association between COVID-19 lockdown and PTB was estimated using the Odds Ratio (OR) obtained from log binomial regression and the Absolute Risk Difference (ARD) obtained from identity link binomial regression. In both groups, the IPTW weights were used with a quasibinomial model to obtain robust variance estimates. To avoid convergence issues, the R package glm2 was used. Group differences were assessed by calculating IPTW proportions, weighted medians, and standardized mean differences.

The differences in the distribution of maternal characteristics between lockdown and non-lockdown groups were evaluated by the chi-square test for categorical variables and the Mann–Whitney *U*-test for continuous variables. Time to delivery was evaluated through Kaplan-Meier estimates, and differences between the groups were tested with a log rank test. Logistic regression was performed to calculate crude and adjusted odds ratios (aORs) and 95% confidence intervals (CIs). Potential confounders included maternal age, parity, preterm birth history, GDM, PIH, and paternal age. The selection of potential confounders was based on biological plausibility, identification of a variable as a confounder in previous studies, changes in the effect estimate of interest, or reduction in the residual variability of the outcome. Sensitivity analysis was used to evaluate the difference in PTB in each subgroup between the matched lockdown and non-lockdown groups to identify potential bias. A two-sided *P*-value of <0.05 was considered statistically significant. All statistical tests were conducted using SPSS version 24.0 and R version 3.6.

## Results

### Baseline Characteristics

**Figure 1:**
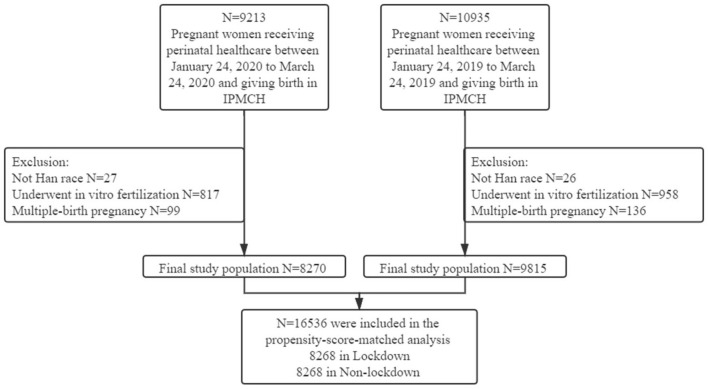
Flow chart of the study population.

A total of 9,213 pregnant women were consecutively enrolled from January 24th, 2020, to March 24th, 2020 (lockdown), and 10935 were enrolled from January 24th, 2019, to March 24th, 2019 (non-lockdown). After the implementation of the exclusion criteria, 8,270 pregnant women in the lockdown group and 9,815 pregnant women in the non-lockdown group remained in the final study population (Figure 1).

Table 1 shows the baseline characteristics between the two groups in the unweighted sample analysis, propensity score-matched analysis, and inverse probability treatment weighting analysis. Compared with the non-lockdown group, women in the lockdown group were more likely to be married (99.4 vs. 98.9%, *P* = 0.001), have a higher BMI (20.8 vs. 20.7 kg/m^2^, *P* = 0.009), have a higher education level (24.2 vs. 21.4%, *P* < 0.001). They were also less likely to be current smokers (0.4 vs. 0.6%, *P* = 0.04), have current alcohol consumption (0.8 vs. 2.6%, *P* < 0.001) and have a preterm birth history (0.2 vs. 0.6%, *P* < 0.001). Through the use of propensity score matching, 8,268 women in the lockdown group were matched with 8,268 women in the non-lockdown group. After matching, the *P*-values of all variables were >0.05, indicating that no significant differences existed between the two groups. Through the use of IPTW, marital status, maternal BMI distribution, smoking and alcohol consumption, and preterm birth history were balanced between the two groups (Table 1).

### Risk of PTB and VPTB in Different Pregnancy Phases

**Figure 2:**
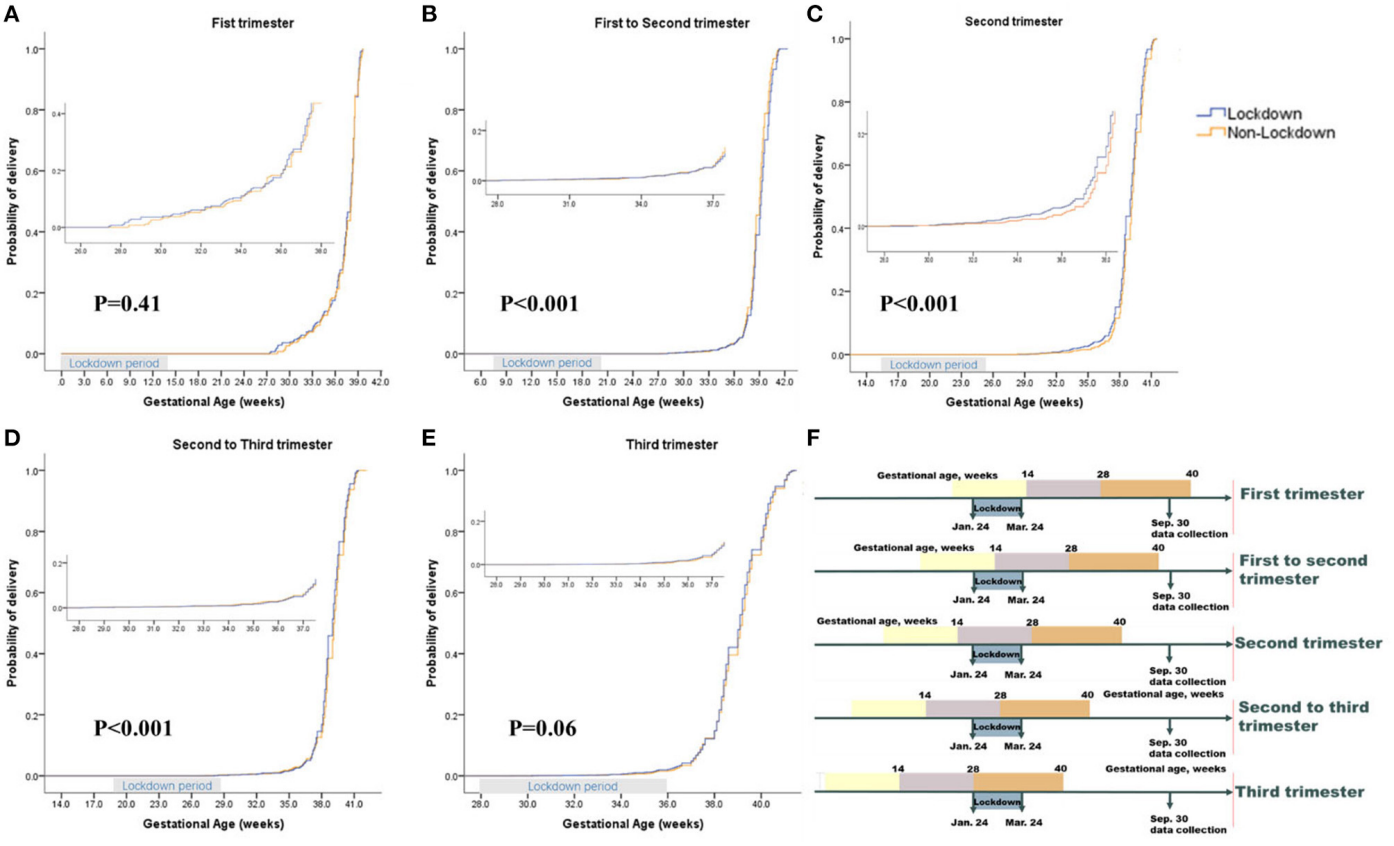
Kaplan-Meier Curve for Lockdown and Non-Lockdown Groups in Propensity-Score-Matched Cohort and the Definition of Different Pregnancy Phases. This figure shows the probability of delivery among pregnant women who were in different pregnancy phases during Lockdown period in two groups. *P*-values were calculated through log rank test. The small picture in each picture is a partial enlarged picture of 28–37 weeks of gestation. **(A)**, Pregnant women who were in First trimester during Lockdown period. **(B)**, Pregnant women who were in First to Second trimester during Lockdown period. **(C)**. Pregnant women who were in Second trimester during Lockdown period. **(D)**. Pregnant women who were in Second to Third trimester during Lockdown period. **(E)**. Pregnant women who were in Third trimester during Lockdown period. **(F)** The definition of different pregnancy phases.

Although there was no group difference in the prevalence of PTB (5.7 vs. 5.3%, *P* = 0.71) or VPTB (1.3 vs. 1.2%, *P* = 0.78) in the total sample (Supplementary Table 1). Women in lockdown during their second trimester had a higher rate of PTB (unweighted sample: 5.9 vs. 4.6%, *P* = 0.08; matched: 5.9 vs. 4.2%, *P* = 0.045; IPTW: 6.0 vs. 4.6%, *P* = 0.02) (Table 2; Figure 2) and a non-significantly higher rate of VPTB (unweighted sample: 1.9 vs. 1.4%, *P* = 0.32; matched: 1.9 vs. 1.2%, *P* = 0.12; IPTW: 2.0 vs. 1.4%, *P* = 0.11) (Table 2) than those in the non-lockdown period. After adjusting for maternal age, parity, GDM, and paternal age, women in their second trimester during lockdown had a higher risk of PTB [unweighted sample: OR: 1.34 (CI 0.97–1.86), ARD: 1.3 (CI 0.3–2.9), *p* = 0.08; matched: OR: 1.43 (CI 1.01–2.02), ARD: 1.7 (CI 0.04–3.4), *p* = 0.045; IPTW: OR: 1.32 (CI 1.05–1.65), ARD: 1.4 (CI 0.3–2.5), *p* = 0.02] (Table 2) and a non-significantly higher risk of VPTB [unweighted sample: OR: 1.33 (CI 0.76–2.32), ARD: 0.5 (CI −0.4 to 1.4), *p* = 0.32; matched: OR: 1.63 (CI 0.85–3.12), ARD: 0.7 (CI −0.2 to 1.6), *P* = 0.14; IPTW: OR: 1.37 (CI 0.93–2.03), ARD: 0.6 (CI −0.05 to 1.3), *p* = 0.11] than those in non-lockdown cohort (Table 2). There were no significant differences between the two groups in PTB or VPTB rates during the other pregnancy phases (first, first to second, second to third and third trimesters) (Supplementary Table 2).

**Table 2:** Analysis of PTB and Subtypes of PTB in Women Exposed in Their Second or Third Trimester.

	**Unweighted Sample**					**Propensity 1:1 Matching**					**Inverse probability of treatment weighting**				
	**No. (AR, %)**		**ARD, % (96% CI)**	**OR (95%CI)**	***P*** **-value**	**No. (AR, %)**		**ARD, % (96% CI)**	**OR (95% CI)**	***P*** **-value**	**No. (AR, %)**		**ARD, % (96% CI)**	**OR (95% CI)**	***P*** **-value**
	**Lock** **down**	**Non-lock** **down**				**Lock** **down**	**Non-lock** **down**				**Lock** **down**	**Non-lock** **down**			
**Second trimester**															
PTB	78 (5.9)	79 (4.6)	1.3 (−0.3 to 2.9)	1.34 (0.97–1.86)	0.08	78 (5.9)	58 (4.2)	1.7 (0.04–3.4)	1.43 (1.01–2.02)	0.04	172 (6.0)	146 (4.6)	1.4 (0.3–2.5)	1.32 (1.05–1.65)	0.02
VPTB	25 (1.9)	25 (1.4)	0.5 (−0.4 to 1.4)	1.33 (0.76–2.32)	0.32	25 (1.9)	16 (1.2)	0.7 (−0.2 to 1.6)	1.63 (0.85–3.12)	0.14	57 (2.0)	46 (1.4)	0.6 (−0.05 to 1.3)	1.37 (0.93–2.03)	0.11
PROM-PTB	33 (2.5)	30 (1.7)	0.8 (−0.2 to 1.8)	1.47 (0.89–2.42)	0.13	33 (2.5)	25 (1.8)	0.7 (−0.4 to 1.8)	1.25 (0.73–2.15)	0.42	72 (2.5)	55 (1.7)	0.8 (0.07–1.5)	1.42 (0.99–2.03)	0.06
S-PTB	21 (1.6)	21 (1.2)	0.4 (−0.5 to 1.3)	1.33 (0.72–2.44)	0.36	21 (1.6)	12 (0.9)	0.7 (−0.1 to 1.5)	1.81 (0.87–3.76)	0.11	49 (1.7)	38 (1.2)	0.5 (−0.1 to 1.1)	1.43 (0.93–2.19)	0.10
MI-PTB	24 (1.8)	28 (1.6)	0.2 (−0.7 to 1.1)	1.14 (0.66–1.97)	0.65	24 (1.8)	21 (1.5)	0.3 (−0.7 to 1.3)	1.15 (0.63–2.12)	0.65	89 (3.1)	86 (2.7)	0.4 (−0.4 to 1.2)	1.14 (0.85–1.55)	0.38
**Third trimester**															
PTB	110 (4.2)	104 (3.5)	0.7 (−0.3 to 1.7)	1.18 (0.90–1.56)	0.23	110 (4.2)	90 (3.5)	0.7 (−0.3 to 1.7)	1.18 (0.89–1.58)	0.25	237 (4.1)	192 (3.5)	0.6 (−0.1 to 1.3)	1.19 (0.98–1.44)	0.08
VPTB	12 (0.5)	11 (0.4)	0.1 (−0.3 to 0.5)	1.21 (0.53–2.75)	0.65	12 (0.5)	10 (0.4)	0.1 (−0.3 to 0.5)	0.98 (0.41–2.32)	0.96	25 (0.4)	21 (0.4)	0 (−0.2 to 0.2)	1.14 (0.64–2.04)	0.66
PROM-PTB	61 (2.3)	45 (1.5)	0.8 (0.08–1.5)	1.52 (1.03–2.24)	0.04	61 (2.3)	37 (1.4)	0.9 (0.2–1.6)	1.64 (1.09–2.47)	0.02	130 (2.3)	83 (1.5)	0.8 (0.3–1.3)	1.51 (1.14–1.99)	0.003
S-PTB	18 (0.7)	25 (0.9)	−0.2 (−0.7 to 0.3)	0.80 (0.43–1.47)	0.47	18 (0.7)	21 (0.8)	−0.1 (−0.6 to 0.4)	0.84 (0.44–1.59)	0.59	38 (0.7)	45 (0.8)	−0.1 (−0.4 to 0.2)	0.81 (0.52–1.24)	0.33
MI-PTB	31 (1.2)	34 (1.2)	0 (−0.6 to 0.6)	1.01 (0.62–1.65)	0.96	31 (2.0)	32 (1.8)	0.2 (−0.5 to 0.9)	0.32 (0.56–1.54)	0.78	121 (2.1)	106 (1.9)	0.2 (−0.3 to 0.7)	1.09 (0.84–1.42)	0.51

*AR, Absolute Risk; ARD, Absolute Risk Difference; OR, Odds Ratio; PTB, Preterm Birth; VPTB, Very Preterm Birth; PROM-PTB, preterm birth with premature rupture of membranes; S-PTB, spontaneous preterm birth with intact membranes; MI-PTB, medically induced preterm birth*.

### The Association Between COVID-19 Lockdown and Subtypes of PTB in Different Pregnancy Phases

We further explored the risk of different clinical subtypes of PTB (PROM-PTB, S-PTB, and MI-PTB) associated with COVID-19 lockdown in different pregnancy phases. The prevalence of PROM-PTB in the third trimester among women in lockdown was higher than that among those in non-lockdown (unweighted sample: 2.3 vs. 1.5%, *P* = 0.04; matched: 2.3 vs. 1.4%, *P* = 0.02; IPTW: 2.3 vs. 1.5%, *P* = 0.003) (Table 2; Supplementary Figure 1). After adjusting for maternal and paternal age and parity, among pregnant women in their third trimester, those in lockdown had an increased risk of PROM-PTB compared to those in non-lockdown [unweighted sample: OR: 1.52 (CI 1.03–2.24), ARD: 0.8 (CI 0.08–1.5), *P* = 0.04; matched: OR: 1.64 (CI 1.09–2.47), ARD: 0.9 (CI 0.2–1.6), *p* = 0.02; IPTW: OR: 1.51 (CI 1.14–1.99), ARD: 0.8 (CI 0.3–1.3), *P* = 0.003] (Table 2). The pregnant women in lockdown during their third trimester did not have an increased risk of S-PTB or MI-PTB in the three types of analysis (Table 2). There were no significant differences between the two groups in PROM-PTB, S-PTB, or MI-PTB rates in the other pregnancy phases (first, first to second, second and second to third trimesters) (Supplementary Figure 1; Supplementary Table 3).

### Sensitivity Analyses

In sensitivity analyses, we divided pregnant women during their second trimester into a propensity score-matched cohort based on whether they had GDM, which resulted in 1,195 pregnant women without GDM and 172 with GDM in the non-lockdown group and 1,102 women without GDM and 211 with GDM in the lockdown group. The association between lockdown and PTB did not change substantially (Supplementary Table 4; Supplementary Figure 2). After adjusting for maternal and paternal age, and parity, pregnant women without or with GDM in lockdown had a higher risk of PTB [without GDM: OR: 1.27 (CI 0.86–1.88), *p* = 0.24; with GDM: OR: 1.73 (CI 0.68–4.40), *p* = 0.25] and VPTB [without GDM: OR: 1.55 (CI 0.77–3.11), *p* = 0.22; with GDM: OR: 3.27 (CI 0.46–23.28), *p* = 0.24] than those in non-lockdown, although the results did not reach statistical significance (Supplementary Table 4). Similar results were also found in the unweighted sample and through the use of IPTW (Supplementary Table 4). We also performed sensitivity analyses for the association between COVID-19 lockdown and PROM-PTB rates among pregnant women with or without GDM during their third trimester in the propensity score-matched cohort. Pregnant women in lockdown during their third trimester, with or without GDM, had a higher risk of PROM-PTB [without GDM: OR: 1.70 (CI 1.08–2.69), *p* = 0.02; with GDM: OR: 1.05 (CI 0.37–2.97), *p* = 0.94] than those in non-lockdown in the propensity score-matched analysis. Similar results were found in the unweighted sample and through the use of IPTW (Supplementary Table 5; Supplementary Figure 3).

## Discussion

This is the first study to examine the relationship between the COVID-19 lockdown and preterm birth among women who were affected by the lockdown in different trimesters. We found that exposure to the COVID-19 lockdown did not reduce, but increased the risk of PTB among pregnant women in their second trimester and increased the risk of PROM-PTB among those in their third trimester.

Previous evidence suggests that the COVID-19 pandemic and the lockdown measures taken by governments to mitigate its impact on population health were associated with reductions in PTBs (8–10, 19). However, these studies focused on the outcomes of pregnant women who were in their third trimester during the COVID-19 lockdown. In our study, we found that the PTB rate was stable among women in their third trimester, which is consistent with a retrospective study conducted in the United Kingdom (20). The difference with previous studies may be attributed to race and the national attitude toward the COVID-19 pandemic and need for lockdown measures. We further explored the impact of the COVID-19 lockdown on women during early or middle pregnancy and found that exposure to the lockdown led to a 1.42-fold risk of PTB among women in the second trimester and a 1.64-fold risk of PROM-PTB among women in the third trimester. Lockdown measures have a significant societal impact, as they lead to an increased risk for unemployment, low-income status, and family conflicts (21). These factors increase a pregnant woman's risk for anxiety and depression (21). Anxiety has been shown to enhance hypothalamus-pituitary-adrenal cortex (HPA) axis activity (22, 23). Studies have demonstrated that the levels of corticotropin-releasing hormone (CRH), which is released when anxiety stimulates the HPA axis, are significantly higher in women who have delivered a preterm infant than in those who gave birth at term (24). Alternatively, the increase in PTB rates among women exposed to the lockdown may have resulted from indirect effects such as reluctance to go to the hospital when needed (e.g., reduced fetal movements or mild vaginal infection) due to a fear of developing a COVID-19 infection or not wanting to increase the burden on the health care system (20). Changes in obstetrical care may also have played a role in the higher rates of PTB due to staff shortages and reduced number of antenatal visits, ultrasound scans, and screening procedures (25). It was consistent with the finding of a systematic review, in which the incidence of preterm birth occurred was similar among pregnant women who tested positive compared with negative for SARS-CoV-2 (26). The preterm birth rates in this review were thought influenced by transient changes in obstetric management (26). Other possible explanations for our findings include (1) changes in referral methods where more high-risk women were sent to the participating hospital or (2) more high-risk women choosing to deliver at the larger more specialized hospital (20). It is important to provide quality obstetric services that also included the effective management of anxiety and depression to try and mitigate the risk for preterm birth.

Our study has numerous strengths. This study provides the first evidence that the effects of the COVID-19 lockdown on the preterm birth rate have a trimester difference. Second, we used several statistical methods (unweighted sample, propensity score matching, and IPTW) to examine the association between exposure to the COVID-19 lockdown and risk for a PTB to ensure the accuracy of the study results. Some limitations also exist. First, we did not confirm the mental health status of participants during the COVID-19 lockdown. However, another study by our team evaluated the mental health of pregnant women in 25 hospitals in China before and during the pandemic and found significantly higher rates of depression and anxiety among those who were pregnant during the COVID-19 lockdown (23). Second, some pregnant women in their early pregnancy during the COVID-19 pandemic had not yet given birth when we completed the data analysis. As such, the sample size for those in their first trimester was slightly smaller. Furthermore, the number of gestational weeks for women examined in their first trimester was lower than that of those examined in other trimesters. Thus, those examined in their first trimester who had given birth and were included in our analyses were naturally more likely to have delivered a preterm infant. This explains why the PTB rate among women examined in their first trimester was higher than that among those examined later in their pregnancy. Last, although we controlled for as many PTB confounding factors as possible, there may be other unmeasured confounders that could have caused bias in our propensity score-matched cohort.

This is the first study to explore the relationship between COVID-19 lockdown and PTB among non-infected women in different trimesters. Different from previous research conclusions, our study suggests that women in their second trimester during the COVID-19 lockdown were at an increased risk for PTB and that those in their third trimester were at an increased risk for PROM-PTB. Sufficient prenatal care and education program should be emphasized for pregnant women during any society lockdown and warrant additional research.

## Data Availability Statement

The raw data supporting the conclusions of this article will be made available by the authors, without undue reservation.

## Ethics Statement

The studies involving human participants were reviewed and approved by International Peace Maternity and Child Health Hospital review board (No. GKLW2019-51). The patients/participants provided their written informed consent to participate in this study.

## Author Contributions

H-fH and Y-tW devised the study concept and design. T-tL and CZ drafted the manuscript and conducted the statistical analysis. LC, LJ, X-hL, and J-xP were responsible for the data collection and quality control. Y-tW, H-fH, C-LD, and BM critically revised the manuscript for important intellectual content. All authors provided administrative, technical, and material support and agreed with the final version of the article.

## Funding

This research was supported by the National Key Research and Development Program of China (2018YFC1002804), National Natural Science Foundation of China (82001571 and 81671412), Program of Shanghai Academic Research Leader (20XD1424100), Outstanding Youth Medical Talents of Shanghai Rising Stars of Medical Talent Youth Development Program, Science and Technology Innovation Fund of Shanghai Jiao Tong University (YG2019GD04 and YG2020YQ29), COVID-19 Prevention and Control Project of the International Peace Maternity and Child Health Hospital (2020COVID1903 and 2020COVID1904), Clinical Research Plan of Shanghai Shenkang Hospital Development Center (SHDC12018X17 and SHDC12019107), CAMS Innovation Fund for Medical Sciences (2019-12M-5-064), and Clinical Research Project of Shanghai Municipal Health Commission (20184Y0210 and 20184Y0349).

## Conflict of Interest

The authors declare that the research was conducted in the absence of any commercial or financial relationships that could be construed as a potential conflict of interest.

## Publisher's Note

All claims expressed in this article are solely those of the authors and do not necessarily represent those of their affiliated organizations, or those of the publisher, the editors and the reviewers. Any product that may be evaluated in this article, or claim that may be made by its manufacturer, is not guaranteed or endorsed by the publisher.
