# Supplementary material for: COVID-19 Lockdown Increased the Risk of Preterm Birth
Source: Front Med (Lausanne). 2021 Sep 27;8:705943. doi: 10.3389/fmed.2021.705943 (PMC8502851; doi:10.3389/fmed.2021.705943)
Supplement: Supplementary file 1 [file Data_Sheet_1.docx]

| **Supplementary Table 1 Pregnancy Outcomes among All Pregnant Women in Total Sample, Unweighted Sample, Propensity Score-Matched Sample, and Inverse Probability of Treatment-Weighted Sample** | | | | | | | | | | | | | | |
| --- | --- | --- | --- | --- | --- | --- | --- | --- | --- | --- | --- | --- | --- | --- |
|  | **Unweighted Sample** | | |  |  | **Propensity 1:1 Matching** | | |  |  | **Inverse probability of treatment weighting** | | | |
|  | **Lockdown (N=8270)** | **Non-lockdown (N=9815)** | **OR [95%CI]** | **P value** |  | **Lockdown (N=8268)** | **Non-lockdown (N=8268)** | **OR [95%CI]** | **P value** |  | **Lockdown (N=18088)** | **Non-lockdown (N=18084)** | **OR [95%CI]** | **P value** |
| **Primary outcome** | |  |  |  |  |  |  |  |  |  |  |  |  |  |
| PTB (<37 weeks) | 471(5.7) | 539(5.5) | 1.04(0.92-1.18) | 0.55 |  | 471(5.7) | 437(5.3) | 1.08(0.95-1.24) | 0.25 |  | 1033(5.7) | 992(5.5) | 1.04(0.95-1.14) | 0.35 |
| VPTB (<34 weeks) | 108(1.3) | 118(1.2) | 1.09(0.84-1.41) | 0.53 |  | 108(1.3) | 97(1.2) | 1.12(0.85-1.47) | 0.44 |  | 238(1.3) | 220(1.2) | 1.08(0.90-1.30) | 0.40 |
| **Secondary outcomes** | | | | | | | | | | | | | | |
| PTB (<37 weeks) | | | | | | | | | | | | | |  |
| PROM-PTB | 233(2.8) | 245(2.5) | 1.13(0.94-1.36) | 0.18 |  | 233(2.8) | 201(2.4) | 1.16(0.96-1.41) | 0.12 |  | 515(2.8) | 448(2.5) | 1.15(1.02-1.31) | 0.03 |
| S-PTB | 97(1.2) | 118(1.2) | 0.98(0.74-1.28) | 0.86 |  | 97(1.2) | 91(1.1) | 1.07(0.80-1.42) | 0.66 |  | 214(1.2) | 216(1.2) | 0.99(0.82-1.20) | 0.92 |
| MI-PTB | 141(1.7) | 176(1.8) | 1.04(0.87-1.24) | 0.65 |  | 141(1.7) | 145(1.8) | 0.97(0.77-1.23) | 0.81 |  | 563(3.1) | 539(3.0) | 1.05(0.93-1.18) | 0.47 |
| VPBT (<34 weeks) | | | | | | | | | | | | | |  |
| PROM-PTB | 37(34.3) | 41(34.7) | 0.98(0.57-1.70) | 0.94 |  | 37(34.3) | 33(34.0) | 1.01(0.57-1.80) | 0.97 |  | 83(34.7) | 77(34.8) | 1.0(0.68-1.46) | 0.98 |
| S-PTB | 34(31.5) | 31(26.3) | 1.29(0.72-2.30) | 0.39 |  | 34(31.5) | 25(25.8) | 1.32(0.72-2.44) | 0.37 |  | 77(32.4) | 57(25.9) | 1.37(0.91-2.05) | 0.13 |
| MI-PTB | 37(34.3) | 46(39.0) | 0.91(0.54-1.53) | 0.46 |  | 37(34.3) | 39(40.2) | 0.78(0.44-1.37) | 0.38 |  | 141(59.2) | 137(62.3) | 0.88(0.61-1.28) | 0.51 |
| **Pregnancy complications** | | | | | | | | | | | | | |  |
| GDM | 1232(14.9) | 1294(13.2) | 1.15(1.06-1.25) | 0.001 |  | 1231(14.9) | 1042(12.6) | 1.21(1.11-1.33) | <0.001 |  | 2693(14.9) | 2387(13.2) | 1.15(1.08-1.22) | <0.001 |
| PIH | 596(7.2) | 638(6.5) | 1.12(0.99-1.25) | 0.06 |  | 595(7.2) | 538(6.5) | 1.11(0.99-1.26) | 0.08 |  | 1228(6.8) | 1231(6.8) | 1.0(0.92-1.08) | 0.95 |
| **Neonatal outcomes** | | | | | | | | | | | | | | |
| Still birth | 14(0.2) | 17(0.2) | 0.98(0.48-1.98) | 0.95 |  | 14(0.2) | 16(0.2) | 0.88(0.43-1.79) | 0.72 |  | 30(0.2) | 31(0.2) | 0.97(0.59-1.60) | 0.90 |
| Abnormal Apgar score | 165(2.0) | 177(1.8) | 1.11(0.89-1.37) | 0.35 |  | 165(2.0) | 151(1.8) | 1.10(0.88-1.37) | 0.43 |  | 358(2.0) | 329(1.8) | 1.09(0.94-1.27) | 0.27 |
| Fetal distress | 1769(21.4) | 1943(19.8) | 1.10(1.03-1.19) | 0.008 |  | 1769(21.4) | 1711(20.7) | 1.04(0.97-1.12) | 0.27 |  | 3859(21.3) | 3583(19.8) | 1.10(1.04-1.16) | <0.001 |
| LBW | 296(3.6) | 322(3.3) | 1.09(0.93-1.29) | 0.27 |  | 296(3.6) | 272(3.3) | 1.09(0.92-1.29) | 0.31 |  | 651(3.6) | 597(3.3) | 1.09(0.98-1.22) | 0.12 |
| VLBW | 44(0.5) | 49(0.5) | 1.07(0.71-1.60) | 0.76 |  | 44(0.5) | 41(0.5) | 1.07(0.70-1.65) | 0.74 |  | 97(0.5) | 91(0.5) | 1.07(0.80-1.42) | 0.66 |
| Macrosomia | 433(5.2) | 520(5.3) | 0.99(0.87-1.13) | 0.85 |  | 433(5.2) | 429(5.2) | 1.01(0.88-1.16) | 0.89 |  | 952(5.3) | 962(5.3) | 0.99(0.90-1.08) | 0.81 |

OR: Odds Ratio; PTB: Preterm Birth; VPTB: Very Preterm Birth; GDM: Gestational Diabetes Melius; PIH: Pregnancy Induced Hypertension; PROM-PTB: preterm birth with premature rupture of membranes; S-PTB: spontaneous preterm birth with intact membranes; MI-PTB, medically induced preterm birth; LBW: Low birth weight; VLBW: Very Low Birth Weight.

| **Supplementary Table 2 Three Type Analysis of PTB among Women Exposed in their Different Pregnancy Phases** | | | | | | | | | | | |
| --- | --- | --- | --- | --- | --- | --- | --- | --- | --- | --- | --- |
| **PTB** | | | | | |  | **VPTB** | | | | |
| **Type Analysis** | **No.(Absolute Risk,%)** | | **Absolute Risk Difference,%(96% CI)** | **Odds Ratio(95%CI)** | **P value** |  | **No.(Absolute Risk,%)** | | **Absolute Risk Difference,%(96% CI)** | **Odds Ratio(95%CI)** | **P value** |
|  | **Lockdown** | **Non-lockdown** |  |  |  |  | **Lockdown** | **Non-lockdown** |  |  |  |
| **Unweighted sample** | | | | | | | | | | | |
| First | 77(27.5) | 84(25.8) | 1.7(-5.4 - 8.8) | 1.09(0.76-1.56) | 0.65 |  | 29(10.4) | 30(9.2) | 1.2(-3.5 - 5.9) | 1.14(0.66-1.95) | 0.64 |
| First to Second | 109(5.2) | 128(5.3) | -0.1(-1.4 - 1.2) | 0.98(0.75-1.27) | 0.87 |  | 25(1.2) | 27(1.1) | 0.1(-0.5 - 0.7) | 1.07(0.62-1.84) | 0.82 |
| Second | 78(5.9) | 79(4.6) | 1.3(-0.3 - 2.9) | 1.34(0.97-1.86) | 0.08 |  | 25(1.9) | 25(1.4) | 0.5(-0.4 - 1.4) | 1.33(0.76-2.32) | 0.32 |
| Second to Third | 97(5.0) | 144(6.0) | -1(-2.4 - 0.4) | 0.83(0.64-1.08) | 0.16 |  | 17(0.9) | 25(1.0) | -0.1(-0.7 - 0.5) | 0.84(0.45-1.57) | 0.59 |
| Third | 110(4.2) | 104(3.5) | 0.7(-0.3 - 1.7) | 1.18(0.90-1.56) | 0.23 |  | 12(0.5) | 11(0.4) | 0.1(-0.3 - 0.5) | 1.21(0.53-2.75) | 0.65 |
| **Matched** | | | | | | | | | | | |
| First | 77(27.5) | 71(26.5) | 1.0(-6.4 - 8.4) | 1.06(0.73-1.55) | 0.76 |  | 29(10.4) | 25(9.3) | 1.1(-3.9 - 6.1) | 1.12(0.64-1.97) | 0.70 |
| First to Second | 109(5.2) | 108(5.2) | 0(-1.3 - 1.3) | 0.92(0.69-1.21) | 0.55 |  | 25(1.2) | 25(1.2) | 0(-0.7 - 0.7) | 0.91(0.52-1.61) | 0.75 |
| Second | 78(5.9) | 58(4.2) | 1.7(0.04-3.4) | 1.43(1.01-2.02) | 0.04 |  | 25(1.9) | 16(1.2) | 0.7(-0.2 - 1.6) | 1.63(0.85-3.12) | 0.14 |
| Second to Third | 97(5.0) | 110(5.6) | -0.6(-2.0 - 0.80) | 0.85(0.64-1.13) | 0.26 |  | 17(0.9) | 21(1.1) | -0.2(-0.8 - 0.4) | 0.81(0.42-1.56) | 0.54 |
| Third | 110(4.2) | 90(3.5) | 0.7(-0.3 - 1.7) | 1.18(0.89-1.58) | 0.25 |  | 12(0.5) | 10(0.4) | 0.1(-0.3 - 0.5) | 0.98(0.41-2.32) | 0.96 |
| **Inverse probability of treatment weighting** | | | | | | | | | | | |
| First | 168(27.1) | 154(26.0) | 1.1(-3.9 - 6.1) | 1.06(0.82-1.36) | 0.67 |  | 63(10.2) | 56(9.4) | 0.8(-2.5 - 4.1) | 1.09(0.74-1.58) | 0.67 |
| First to Second | 241(5.2) | 234(5.3) | -0.1(-1 - 0.8) | 0.98(0.81-1.17) | 0.79 |  | 57(1.2) | 50(1.1) | 0.1(-0.3 - 0.5) | 1.08(0.74-1.59) | 0.69 |
| Second | 172(6.0) | 146(4.6) | 1.4(0.3 - 2.5) | 1.32(1.05-1.65) | 0.02 |  | 57(2.0) | 46(1.4) | 0.6(-0.05 - 1.3) | 1.37(0.93-2.03) | 0.11 |
| Second to Third | 215(5.1) | 265(5.9) | -0.8(-1.8 - 0.2) | 0.85(0.70-1.02) | 0.08 |  | 36(0.9) | 47(1.1) | -0.2(-0.6 - 0.2) | 0.80(0.52-1.24) | 0.33 |
| Third | 237(4.1) | 192(3.5) | 0.6(-0.1 - 1.3) | 1.19(0.98-1.44) | 0.08 |  | 25(0.4) | 21(0.4) | 0(-0.2 - 0.2) | 1.14(0.64-2.04) | 0.66 |

| **Supplementary Table 3 Three Type Analysis of Subtypes of PTB in Women Exposed during Different Gestational Weeks** | | | | | | | | | | | | | | | | | |
| --- | --- | --- | --- | --- | --- | --- | --- | --- | --- | --- | --- | --- | --- | --- | --- | --- | --- |
| **PROM-PTB** | | | | | |  | **S-PTB** | | | | |  | **MI-PTB** | | | | |
| **Type Analysis** | **No.(AR,%)** | | **ARD,%(96% CI)** | **OR(95%CI)** | **P value** |  | **No.(AR,%)** | | **ARD,%(96% CI)** | **OR(95%CI)** | **P value** |  | **No.(AR,%)** | | **ARD,%(96% CI)** | **OR(95%CI)** | **P value** |
|  | **Lockdown** | **Non-lockdown** |  |  |  |  | **Lockdown** | **Non-lockdown** |  |  |  |  | **Lockdown** | **Non-lockdown** |  |  |  |
| **Unweighted sample** | | | | | | | | | | | | | | | | | |
| First | 34(12.1) | 36(11.1) | 1(-4.1 - 6.1) | 1.11(0.67-1.83) | 0.68 |  | 17(6.1) | 24(7.4) | -1.3(-5.3 - 2.7) | 0.81(0.43-1.54) | 0.52 |  | 26(9.3) | 24(7.4) | 1.9(-2.5 - 6.3) | 1.28(0.72-2.29) | 0.40 |
| First to Second | 52(2.5) | 63(2.6) | -0.1(-1.0 - 0.8) | 0.95(0.65-1.38) | 0.78 |  | 29(1.4) | 23(1.0) | 0.4(-0.2 - 1.0) | 1.46(0.84-2.53) | 0.18 |  | 28(1.3) | 42(1.7) | -0.4(-1.1 - 0.3) | 0.76(0.47-1.24) | 0.27 |
| Second | 33(2.5) | 30(1.7) | 0.8(-0.2 - 1.8) | 1.47(0.89-2.42) | 0.13 |  | 21(1.6) | 21(1.2) | 0.4(-0.5 - 1.3) | 1.33(0.72-2.44) | 0.36 |  | 24(1.8) | 28(1.6) | 0.2(-0.7 - 1.1) | 1.14(0.66-1.97) | 0.65 |
| Second to Third | 53(2.7) | 71(2.9) | -0.2(-1.2 - 0.8) | 0.93(0.65-1.33) | 0.68 |  | 12(0.6) | 25(1.0) | -0.4(-0.9 - 0.1) | 0.59(0.30-1.19) | 0.14 |  | 32(1.6) | 48(2.0) | -0.4(-1.2 - 0.4) | 0.83(0.53-1.30) | 0.41 |
| Third | 61(2.3) | 45(1.5) | 0.8(0.08 - 1.5) | 1.52(1.03-2.24) | 0.04 |  | 18(0.7) | 25(0.9) | -0.2(-0.7 - 0.3) | 0.80(0.43-1.47) | 0.47 |  | 31(1.2) | 34(1.2) | 0(-0.6 - 0.6) | 1.01(0.62-1.65) | 0.96 |
| **Matched** | | | | | | | | | | | | | | | | | |
| First | 34(12.1) | 31(11.6) | 0.5(-4.9 - 5.9) | 1.06(0.63-1.78) | 0.83 |  | 17(6.1) | 20(7.5) | -1.4(-5.6 - 2.8) | 0.83(0.42-1.62) | 0.58 |  | 26(9.3) | 20(7.5) | 1.8(-2.8 - 6.4) | 1.20(0.64-2.26) | 0.57 |
| First to Second | 52(2.5) | 54(2.6) | -0.1(-1.1 - 0.9) | 0.88(0.60-1.31) | 0.54 |  | 29(1.4) | 19(0.9) | 0.5(-0.1 - 1.1) | 1.41(0.78-2.55) | 0.26 |  | 28(1.3) | 35(1.7) | -0.4(-1.1 - 0.3) | 0.74(0.44-1.24) | 0.25 |
| Second | 33(2.5) | 25(1.8) | 0.7(-0.4 - 1.8) | 1.25(0.73-2.15) | 0.42 |  | 21(1.6) | 12(0.9) | 0.7(-0.1 - 1.5) | 1.81(0.87-3.76) | 0.11 |  | 24(1.8) | 21(1.5) | 0.3(-0.7 - 1.3) | 1.15(0.63-2.12) | 0.65 |
| Second to Third | 53(2.7) | 54(2.7) | 0(-1.0 - 1.0) | 0.97(0.65-1.43) | 0.87 |  | 12(0.6) | 19(1.0) | -0.4(-1.0 - 0.2) | 0.61(0.29-1.29) | 0.19 |  | 32(1.6) | 37(1.9) | -0.3(-1.1 - 0.5) | 0.81(0.50-1.31) | 0.38 |
| Third | 61(2.3) | 37(1.4) | 0.9(0.2 - 1.6) | 1.58(1.04-2.40) | 0.03 |  | 18(0.7) | 21(0.8) | -0.1(-0.6 - 0.4) | 0.84(0.44-1.59) | 0.59 |  | 31(2.0) | 32(1.8) | 0.2(-0.5 - 0.9) | 0.32(0.56-1.54) | 0.78 |
| **Inverse probability of treatment weighting** | | | |  |  |  |  |  |  |  |  |  |  |  |  |  |  |
| First | 75(12.1) | 66(11.1) | 1(-2.6 - 4.6) | 1.10(0.77-1.56) | 0.61 |  | 37(6.0) | 44(7.4) | -1.4(-4.2 - 1.4) | 0.79(0.50-1.24) | 0.31 |  | 111(17.9) | 87(14.7) | 3.2(-1.0 - 7.4) | 1.27(0.93-1.72) | 0.13 |
| First to Second | 117(2.5) | 116(2.6) | -0.1(-0.8 - 0.6) | 0.96(0.74-1.24) | 0.73 |  | 63(1.4) | 42(1.0) | 0.4(-0.05 - 0.8) | 1.43(0.96-2.12) | 0.07 |  | 125(2.7) | 128(2.9) | -0.2(-0.9 - 0.5) | 0.92(0.72-1.19) | 0.53 |
| Second | 72(2.5) | 55(1.7) | 0.8(0.07 - 1.5) | 1.42(0.99-2.03) | 0.06 |  | 49(1.7) | 38(1.2) | 0.5(-0.1 - 1.1) | 1.43(0.93-2.19) | 0.1 |  | 89(3.1) | 86(2.7) | 0.4(-0.4 - 1.2) | 1.14(0.85-1.55) | 0.38 |
| Second to Third | 121(2.9) | 129(2.9) | 0(-0.7 - 0.7) | 0.99(0.77-1.27) | 0.92 |  | 26(0.6) | 46(1.0) | -0.4(-0.8- -0.03) | 0.59(0.37-0.96) | 0.03 |  | 117(2.8) | 132(3.0) | -0.2(-0.9 - 0.5) | 0.93(0.72-1.20) | 0.58 |
| Third | 130(2.3) | 83(1.5) | 0.8(0.3 - 1.3) | 1.51(1.14-1.99) | 0.003 |  | 38(0.7) | 45(0.8) | -0.1(-0.4 - 0.2) | 0.81(0.52-1.24) | 0.33 |  | 121(2.1) | 106(1.9) | 0.2(-0.3 - 0.7) | 1.09(0.84-1.42) | 0.51 |

AR: Absolute Risk, ARD: Absolute Risk Difference; OR: Odds Ratio; PTB: Preterm Birth; VPTB: Very Preterm Birth; PROM-PTB: preterm birth with premature rupture of membranes; S-PTB: spontaneous preterm birth with intact membranes; MI-PTB, medically induced preterm birth.

| **Supplementary Table 4 Analysis of Risk of Preterm Birth among Women Exposed during their Second Trimester, Sub-analyzed by with or without GDM** | | | | | | | | | | | | | | |
| --- | --- | --- | --- | --- | --- | --- | --- | --- | --- | --- | --- | --- | --- | --- |
| **Type Analysis** | **Total** | | | |  | **Without GDM** | | | |  | **With GDM** | | | |
|  | **N(%)** | | **Adjusted OR[95%CI]** | **P value** |  | **N(%)** | | **Adjusted OR[95%CI]** | **P value** |  | **N(%)** | | **Adjusted OR[95%CI]** | **P value** |
|  | **Lockdown** | **Non-lockdown** |  |  |  | **Lockdown** | **Non-lockdown** |  |  |  | **Lockdown** | **Non-lockdown** |  |  |
| PTB | | | | | | | | | | | | | | |
| Unweighted Sample | 78(5.9) | 79(4.6) | 1.34(0.97-1.86) | 0.08 |  | 61(5.5) | 64(4.3) | 1.32(0.92-1.88) | 0.14 |  | 17(8.1) | 15(6.4) | 1.29(0.63-2.64) | 0.49 |
| Matched | 78(5.9) | 58(4.2) | 1.43(1.01-2.02) | 0.04 |  | 61(5.5) | 50(4.2) | 1.27(0.86-1.88) | 0.24 |  | 17(8.1) | 8(4.7) | 1.73(0.68-4.40) | 0.25 |
| Inverse probability of treatment weighting | 172(6.0) | 146(4.6) | 1.32(1.05-1.65) | 0.02 |  | 136(5.6) | 119(4.3) | 1.31(1.02-1.69) | 0.03 |  | 37(8.0) | 28(6.5) | 1.25(0.75-2.08) | 0.39 |
| VPTB | | | | | | | | | | | | | | |
| Unweighted Sample | 25(1.9) | 25(1.4) | 1.33(0.76-2.32) | 0.32 |  | 21(1.9) | 20(1.3) | 1.44(0.78-2.67) | 0.25 |  | 4(1.9) | 5(2.1) | 0.89(0.24-3.36) | 0.86 |
| Matched | 25(1.9) | 16(1.2) | 1.63(0.85-3.12) | 0.14 |  | 21(1.9) | 14(1.2) | 1.55(0.77-3.11) | 0.22 |  | 4(1.9) | 2(1.2) | 3.27(0.46-23.28) | 0.24 |
| Inverse probability of treatment weighting | 57(2.0) | 46(1.4) | 1.37(0.93-2.03) | 0.11 |  | 49(2.0) | 37(1.3) | 1.51(0.98-2.33) | 0.06 |  | 9(1.9) | 9(2.1) | 0.93(0.37-2.36) | 0.87 |

| **Supplementary Table 5 Analysis of Risk of Subtypes of PROM-PTB among Women Exposed during their Third Trimester, Sub-analyzed by with or without GDM** | | | | | | | | | | | | | | |
| --- | --- | --- | --- | --- | --- | --- | --- | --- | --- | --- | --- | --- | --- | --- |
| **Type Analysis** | **Total** | | | |  | **Without GDM** | | | |  | **With GDM** | | | |
|  | **N(%)** | | **Adjusted OR[95%CI]** | **P value** |  | **N(%)** | | **Adjusted OR[95%CI]** | **P value** |  | **N(%)** | | **Adjusted OR[95%CI]** | **P value** |
|  | **Lockdown** | **Non-lockdown** |  |  |  | **Lockdown** | **Non-lockdown** |  |  |  | **Lockdown** | **Non-lockdown** |  |  |
| Unweighted Sample | 61(2.3) | 45(1.5) | 1.52(1.03-2.24) | 0.04 |  | 53(2.3) | 36(1.4) | 1.64(1.07-2.51) | 0.02 |  | 8(2.7) | 9(2.6) | 1.05(0.40-2.76) | 0.92 |
| Matched | 61(2.3) | 37(1.4) | 1.58(1.04-2.40) | 0.03 |  | 53(2.3) | 30(1.3) | 1.70(1.08-2.69) | 0.02 |  | 8(2.7) | 7(2.4) | 1.05(0.37-2.97) | 0.94 |
| Inverse probability of treatment weighting | 130(2.3) | 83(1.5) | 1.51(1.14-1.99) | 0.003 |  | 113(2.2) | 67(1.4) | 1.61(1.19-2.19) | 0.002 |  | 17(2.7) | 16(2.5) | 1.10(0.55-2.19) | 0.8 |

**
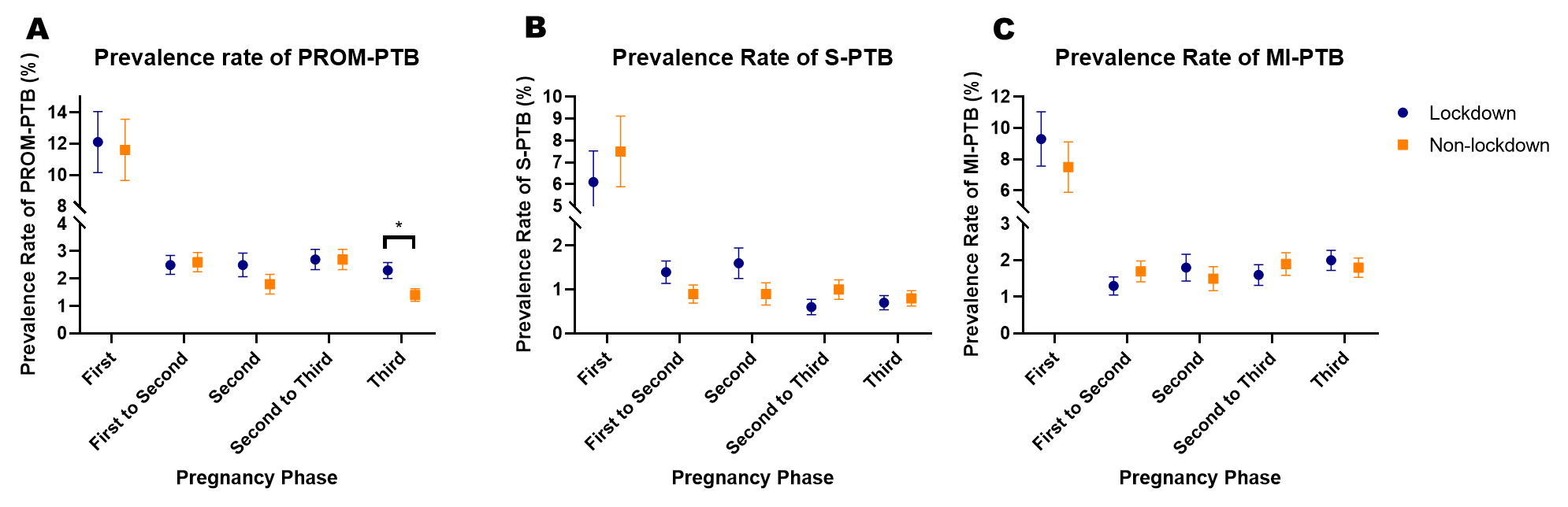
**

**Supplementary Figure 1 The prevalence of subtypes of preterm birth among women exposed in their different pregnancy phases between the propensity-score-matched groups.** A, Prevalence rate of PROM-PTB. B, Prevalence rate of S-PTB. C, Prevalence rate of MI-PTB. First: pregnant women who were in their first trimester during the COVID-19 epidemic. First to Second: pregnant women who were in their first trimester when the COVID-19 outbreak, and were in second trimester when the COVID-19 epidemic ended. Second: pregnant women who were in their second trimester during the COVID-19 epidemic. Second to third: pregnant women who were in their second trimester when the COVID-19 outbreak, and were in their third trimester when the COVID-19 epidemic ended. Third: pregnant women who were in their third trimester during the COVID-19 epidemic. PROM-PTB, preterm birth with premature rupture of membranes; S-PTB, spontaneous preterm birth with intact membranes; MI-PTB, medically induced preterm birth. *, P<0.05

**
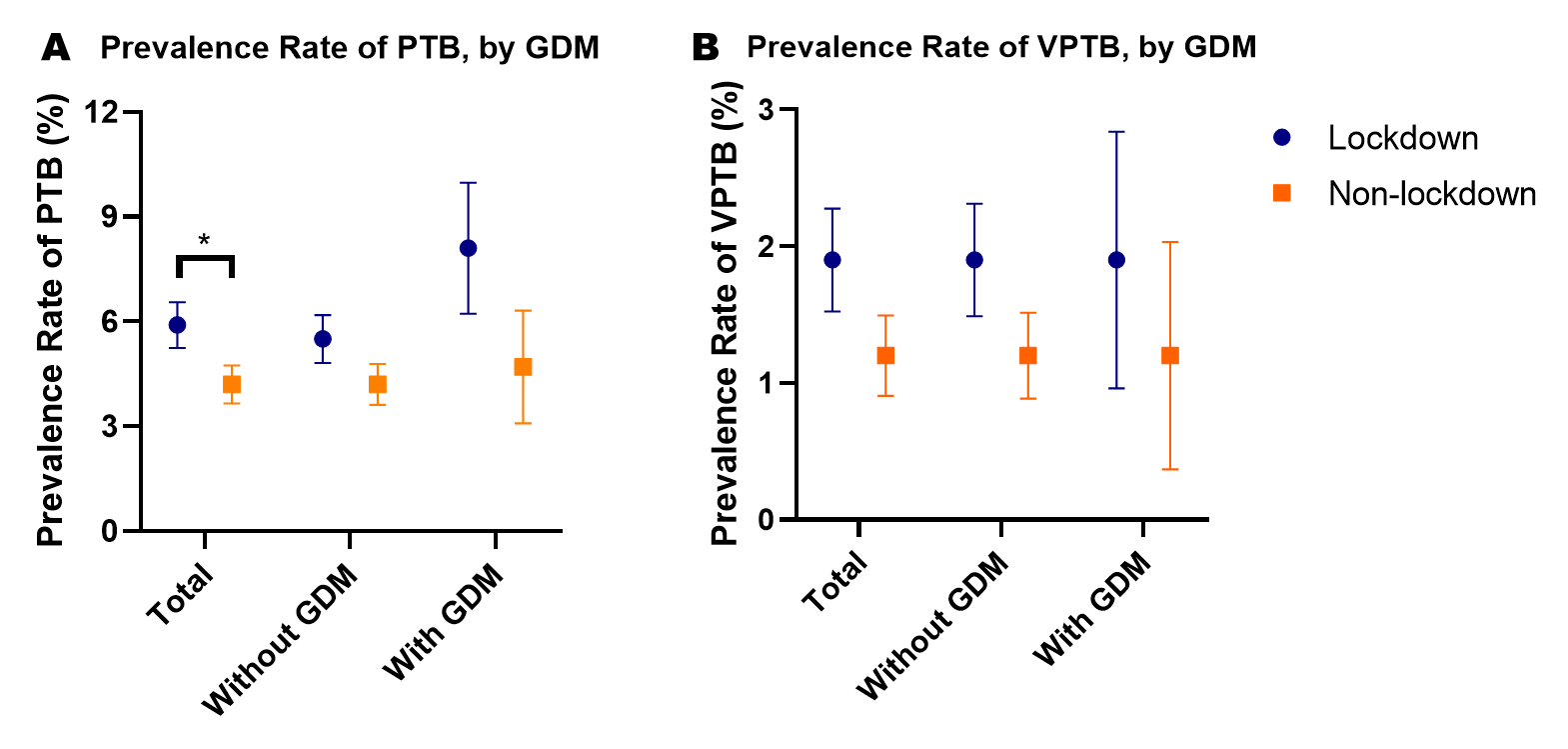
**

**Supplementary Figure 2 The prevalence of preterm birth in the second trimester between the propensity-score-matched groups, by GDM.** A, Prevalence rate of PTB. B, Prevalence rate of VPTB. GDM, gestational diabetes mellitus; PTB, preterm birth; VPTB, very preterm birth. *, P<0.05


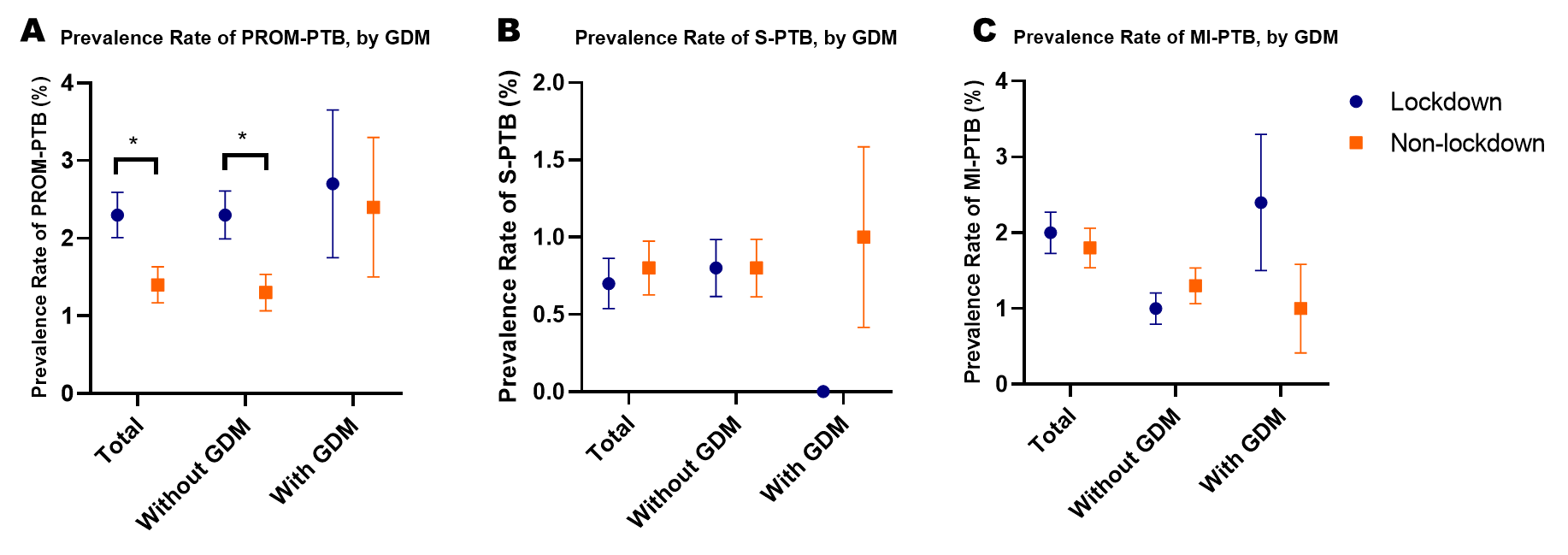


**Supplementary Figure 3 The prevalence of subtypes of preterm birth in the third trimester between the propensity-score-matched groups, by GDM. A,** Prevalence rate of PROM-PTB. **B,** Prevalence rate of S-PTB. **C,** Prevalence rate of MI-PTB. GDM, gestational diabetes mellitus; PROM-PTB, preterm birth with premature rupture of membranes; S-PTB, spontaneous preterm birth with intact membranes; MI-PTB, medically induced preterm birth. *, P<0.05
